# Rethinking the Relationship between Social Experience and False-Belief Understanding: A Mentalistic Account

**DOI:** 10.3389/fpsyg.2016.01721

**Published:** 2016-11-03

**Authors:** Erin Roby, Rose M. Scott

**Affiliations:** School of Social Sciences, Humanities, and Arts, University of California MercedMerced, CA, USA

**Keywords:** false-belief understanding, psychological reasoning, mental-state talk, social cognition, social understanding

## Abstract

It was long assumed that the capacity to represent false beliefs did not emerge until at least age four, as evidenced by children’s performance on elicited-response tasks. However, recent evidence that infants appear to demonstrate false-belief understanding when tested with alternative, non-elicited-response measures has led some researchers to conclude that the capacity to represent beliefs emerges in the 1st year of life. This mentalistic view has been criticized for failing to offer an explanation for the well-established positive associations between social factors and preschoolers’ performance on elicited-response false-belief tasks. In this paper, we address this criticism by offering an account that reconciles these associations with the mentalistic claim that false-belief understanding emerges in infancy. We propose that rather than facilitating the emergence of the capacity to represent beliefs, social factors facilitate the use of this ability via effects on attention, inference, retrieval, and response production. Our account predicts that the relationship between social factors and false-belief understanding should not be specific to preschoolers’ performance in elicited-response tasks: this relationship should be apparent across the lifespan in a variety of paradigms. We review an accumulating body of evidence that supports this prediction.

## Introduction

Many everyday interactions involve predicting and interpreting others’ behavior on the basis of their mental states (e.g., goals, preferences, and beliefs). Researchers have long been interested in when and how the ability to attribute mental states to others develops. In particular, considerable research has focused on when children understand that others can be mistaken, or hold false beliefs, about the world. Traditionally, false-belief understanding was investigated using elicited-response tasks, which require children to answer direct questions about the behavior of a mistaken agent. In one such task (e.g., Baron-Cohen et al., 1985), children hear a story enacted with props: Sally puts a marble in a basket; in her absence, the marble is moved to a nearby box. Children are then asked, “Where will Sally look for her marble?” Beginning around age 4, children correctly say that Sally will look in the basket, where she falsely believes it to be. In contrast, younger children incorrectly respond that Sally will look for the marble in its actual location, suggesting a failure to appreciate Sally’s false belief. This widely replicated finding led many to conclude that children cannot represent false beliefs until at least age 4 (e.g., Wellman et al., 2001).

Recently, however, researchers have developed a variety of alternative non-elicited-response paradigms for assessing false-belief understanding, the results of which suggest that this ability might be present much earlier than previously thought. Positive results have been obtained with children between 6 months and 3 years of age using these alternative measures (for a review, see Scott et al., in press). For instance, infants visually anticipate where a mistaken agent will search for an object (e.g., Southgate et al., 2007; Senju et al., 2011; He et al., 2012; Surian and Geraci, 2012), look reliably longer when an agent’s actions are inconsistent with her false belief (e.g., Onishi and Baillargeon, 2005; Surian et al., 2007; Song and Baillargeon, 2008; Scott and Baillargeon, 2009; Luo, 2011), and use an agent’s false belief to guide their own helping behaviors (e.g., Buttelmann et al., 2009; Knudsen and Liszkowski, 2012) as well as their interpretation of a mistaken agent’s communicative acts (Southgate et al., 2010).

Advocates of *mentalistic* accounts argue that these recent findings demonstrate that false-belief understanding emerges in the 1st year of life (e.g., Leslie, 2005; Southgate et al., 2007; Surian et al., 2007; Buttelmann et al., 2009; Kovács et al., 2010; Luo, 2011; Carruthers, 2013; Baillargeon et al., 2015; Scott et al., in press). However, others have argued that responses in non-elicited-response tasks do not reflect a genuine understanding of belief and are instead driven by more limited, rudimentary capacities, such as low-level perceptual novelty (e.g., Heyes, 2014), learned behavioral rules (e.g., Ruffman, 2014), or a system for tracking belief-like states (e.g., Apperly and Butterfill, 2009). According to these *late-emergence* accounts, false-belief understanding does not emerge until age four, as indicated by success on elicited-response tasks.

The merits of these accounts have been extensively debated elsewhere (e.g., Butterfill and Apperly, 2013; Heyes, 2014; Ruffman, 2014; Scott, 2014; Scott and Baillargeon, 2014; Scott et al., 2015; Carruthers, 2016, in press; Christensen and Michael, 2016; Low et al., 2016; Michael and Christensen, 2016), and we will not repeat those arguments here. Instead, we take up a specific challenge to mentalistic accounts that has yet to be addressed in the literature: the criticism that mentalistic accounts do not address the relationship between social factors and false-belief performance (e.g., San Juan and Astington, 2012; Ruffman, 2014). We first briefly review the evidence for this relationship and then offer a new account that reconciles these findings with the mentalistic claim that false-belief understanding emerges in infancy.

## Social Experience and Preschoolers’ False-Belief Performance

Preschoolers’ performance on elicited-response false-belief tasks is correlated with a variety of social factors (e.g., Perner et al., 1994; Holmes et al., 1996; Dunn and Cutting, 1999; Hughes et al., 1999; Meins et al., 2003; Lecce and Hughes, 2010; Mayer and Träuble, 2013; McAlister and Peterson, 2013; for detailed discussions of these factors and children’s social experience, see Carpendale and Lewis, 2004; Wellman, 2014; Taumoepeau, 2015). For instance, preschoolers from larger families (e.g., Perner et al., 1994) and those with same-aged or older siblings (e.g., McAlister and Peterson, 2013) show superior performance on elicited-response false-belief tasks. There is also a well-established positive relationship between preschoolers’ false-belief performance and mental-state language: terms that refer to psychological states such as *think, know*, and *understand* (Brown et al., 1996; Nielsen and Dissanayake, 2000; Ruffman et al., 2002; Adrián et al., 2005; Ensor and Hughes, 2008; Howard et al., 2008). Parents’ use of mental-state terms in both laboratory settings and home environments predicts their preschoolers’ performance on elicited-response tasks concurrently (e.g., Howard et al., 2008) and longitudinally (e.g., Ruffman et al., 2002). Training that exposes preschoolers to additional mental-talk improves their elicited-response performance (e.g., Lohmann and Tomasello, 2003; Taumoepeau and Reese, 2013). Moreover, deaf children raised by hearing parents, who hear fewer references to mental states than their hearing counterparts, exhibit deficits in elicited-response performance (Gale et al., 1996; Moeller and Schick, 2006; Meristo et al., 2007), as do children from cultures where parents do not typically discuss mental states with their children (Mayer and Träuble, 2013; Taumoepeau, 2015). Preschoolers’ performance on elicited-response tasks is also related to their own personal use of mental-state language in naturalistic settings such as free play with peers (e.g., Brown et al., 1996) and in laboratory settings (e.g., Ruffman et al., 2002).

From the perspective of late-emergence accounts, the reason for these associations is straightforward: certain social experiences facilitate the emergence of the ability to represent false beliefs, which in turn leads to successful performance in elicited-response tasks. Several such explanations have been proposed (Meins et al., 2003; Carpendale and Lewis, 2004; de Villiers, 2005; Nelson, 2005; Ensor and Hughes, 2008; San Juan and Astington, 2012; Ruffman, 2014; Taumoepeau and Ruffman, 2016). For instance, some have suggested that these social experiences both afford children the opportunity to reflect on the mind and confront them with the fact that others’ thoughts and feelings can differ from their own (e.g., Ruffman et al., 1999; Carpendale and Lewis, 2004; Harris, 2005; Ensor and Hughes, 2008); this helps children construct mental-state concepts, including the concept of belief, and link those concepts to behavior (San Juan and Astington, 2012; Ruffman, 2014; Taumoepeau and Ruffman, 2016). Conversations involving mental-state language have been argued to be especially helpful in this regard: by providing explicit labels for mental-state concepts, epistemic verbs such as *think* and *know* help children detect patterns of behavior across disparate situations, thereby leading to an abstract understanding of how mental states translate into action (e.g., San Juan and Astington, 2012). Finally, some have argued that the syntactic structure in which epistemic verbs occur (e.g., sentential complements: She thinks *that it is raining*) is necessary for explicitly representing and reasoning about beliefs (e.g., de Villiers, 2005).

## A Mentalistic Account of this Relationship

Several researchers have criticized mentalistic accounts for not yet providing an explanation for the relationships described above (San Juan and Astington, 2012; Ruffman, 2014). If the capacity to represent beliefs emerges in infancy, as argued by mentalistic accounts, then it cannot be the case that social experience gives rise to this ability. Why then do social factors predict false-belief performance?

We propose that rather than facilitating the emergence of the ability to represent beliefs, social factors facilitate the use of this ability. We assume that the ability to represent beliefs emerges in infancy, as demonstrated by children’s performance on non-elicited-response tasks. However, the capacity to represent beliefs does not guarantee success in elicited- or non-elicited-response tasks: a number of factors mediate between this capacity and successful performance in any given situation (e.g., Lewis and Osborne, 1990; Leslie and Polizzi, 1998; Bloom and German, 2000; Baillargeon et al., 2010; Rubio-Fernández and Geurts, 2013; Helming et al., 2014; Scott and Roby, 2015; Scott et al., in press). Below we outline several such factors and discuss how social experience might interact with these factors to influence children’s false-belief reasoning.

### Attention

In order to infer an agent’s false belief, one must attend to that agent’s behavior and any belief-relevant information within a scene. Social experiences might affect false-belief performance in part by influencing how individuals attend to social situations. Social interactions that focus on other individuals, such as conversations about others’ mental states or play with peers and siblings, might increase interest in agents and their mental states. Individuals who frequently engage in such interactions might be more inclined to attend to agents over other aspects of a scene. Social experience might also encourage individuals to attend selectively to specific aspects of an agent’s behavior that are relevant to inferring that agent’s mental states. For instance, although infants are sensitive to eye contact from birth (Farroni et al., 2002), social experience appears to shape the way in which children attend to and use gaze information: sighted infants of blind parents devote less attention to others’ eyes and to the targets of others’ gaze than do infants of sighted parents, and this difference increases with age (Senju et al., 2015). Finally, events that disrupt attention to agents interfere with children’s and adults’ false-belief performance (Rubio-Fernández, 2013; Rubio-Fernández and Geurts, 2013). Individuals who routinely engage in other-focused interactions might be better at sustaining attention to agents and belief-relevant information and thus be more resistant to such disruptive effects.

### Inference

Attending to an agent does not guarantee that one will successfully infer that agent’s mental states or reason about that agent’s subsequent actions: one might be unable to create a causal model of the events unfolding within the scene due to a lack of situational knowledge (e.g., Christensen and Michael, 2016). To illustrate, consider an item from the Strange Stories task (White et al., 2009): participants read a story in which a nervous woman is walking home at night when a man approaches her to ask the time; the woman says, “Take my purse, just don’t hurt me please!” Participants are asked why she said this. To respond correctly, participants must use contextual cues to infer that the woman falsely believed the man was a robber. School-aged children have difficulty with this and other advanced mental-state reasoning tasks that require them to infer beliefs and desires based on subtle situational cues, even though they are certainly capable of representing such mental states (e.g., Devine and Hughes, 2013; Bianco et al., 2016). Interacting with peers and siblings and discussing others’ mental states might facilitate the acquisition of the situational knowledge needed to build causal models of scenarios such as the one just described (perhaps in the form of schemas, Christensen and Michael, 2016). This in turn would result in an improved ability to infer others’ mental states in a range of situations based on the (sometimes limited) information available (e.g., Bianco et al., 2016).

### Retrieval

Successful false-belief reasoning sometimes requires retrieving from memory information about types of social situations (as just discussed), events that recently occurred in a specific situation, or mental states that one had previously attributed to an agent in that situation. Social interactions might facilitate access to this information via the creation of retrieval cues. In particular, learning and using mental-state terms likely provides an especially useful tool for retrieving belief-relevant information, as well as holding that information in mind while planning a response to an agent or to a question about that agent.

### Responding

Assuming that one successfully attends to, infers, and retrieves an agent’s false belief, social factors might facilitate the ability to generate appropriate responses based on that belief in several ways. First, we have argued that when children are asked the test question in traditional elicited-response false-belief tasks (e.g., “Where will Sally look for her marble?”), this initiates a response-selection process: children must interpret the test question and select an appropriate response (e.g., Baillargeon et al., 2010; Scott and Roby, 2015; Scott et al., in press). This response-selection process often triggers a prepotent bias to respond based on reality, and this response must be inhibited in order to answer based on the agent’s false belief (response-inhibition process). Several sources of difficulty likely contribute to this bias: pragmatic factors might cause children to misinterpret the question as asking where the marble is or where Sally ought to look to find it, mentioning the marble might draw children’s attention to its location, and children’s own knowledge may be naturally more salient (Leslie and Polizzi, 1998; Hansen, 2010; Rubio-Fernández and Geurts, 2013; Helming et al., 2014; Baillargeon et al., 2015). Frequently engaging in social interactions that involve discussion of others’ mental states, especially those involving direct questions (e.g., Howard et al., 2008), might help children overcome pragmatic difficulties and correctly interpret the experimenter’s question. Similarly, social interactions might provide practice inhibiting one’s own beliefs and desires in order to focus on those of another, thereby facilitating correct responding in elicited-response tasks.

Because children are not asked direct questions in non-elicited-response tasks, the response-selection process is not activated and thus no prepotent responses are generated. However, recent evidence suggests that under some circumstances, non-elicited-response tasks might involve some form of inhibition that is unrelated to the response-selection process (e.g., Yott and Poulin-Dubois, 2012; Wang and Leslie, 2016). Social experiences that improve inhibition might therefore facilitate performance in these tasks as well.

Second, consider recent non-elicited-response false-belief tasks in which children are prompted to help a mistaken agent (e.g., Buttelmann et al. 2009, 2014, 2015). In order to assist the agent, not only must children use the agent’s false belief to infer the agent’s likely goal, but they must also (1) understand that the agent needs help, (2) realize that they are capable of helping and choose to do so, (3) determine how they could best assist, and (4) plan and execute that helping response. Successful responding thus requires children to integrate multiple inferences about the agent’s mental states with inferences about their own knowledge and capabilities in order to produce appropriate helping behavior. Social experience likely plays a critical role in developing such integrative abilities.

## Predictions from this Mentalistic Account

The preceding analysis has implications for the relationship between social factors and false-belief understanding. If social factors facilitate the use, rather than the emergence, of mental-state concepts, then there is no reason why the relationship between these factors and false-belief understanding should be specific to preschoolers’ performance on elicited-response tasks: this relationship should be evident across the lifespan in a variety of tasks. This leads to at least two specific predictions regarding the relationship between social experience and false-belief performance.

First, social experience should be related to children’s performance on non-elicited-response false-belief tasks prior to age four. Although these tasks do not require children to answer direct questions about a mistaken agent’s behavior, they require children to attend to, infer, and retrieve an agent’s false beliefs. If social factors facilitate these processes, as we have argued, then these factors should be positively associated with younger children’s performance on non-elicited-response tasks. Consistent with this prediction, Meristo et al. (2012) found that 23-month-old deaf infants raised by hearing parents failed a non-elicited-response false-belief task, whereas hearing infants of hearing parents succeeded. Although Meristo et al. (2012) did not directly examine the social experiences of these infants, recent evidence suggests that interactions between hearing mothers and their deaf infants involve significantly fewer cognitive terms (e.g., *think, know*) and connected turns than do interactions between hearing mothers and hearing infants (Morgan et al., 2014). Given that these factors predict older children’s performance on elicited-response tasks (e.g., Ensor and Hughes, 2008), these findings provide suggestive, albeit indirect, evidence that differences in social experience can affect early false-belief performance.

Ongoing work in our lab provides more direct support for this relationship: parents view a picture book (Taumoepeau and Ruffman, 2006) with their 2.5-year-old child and we measure the percentage of their utterances that contain mental-state terms. Preliminary results indicate that parental use of cognitive terms predicts 2.5-year-olds’ performance in both verbal (Roby and Scott, 2015) and non-verbal (Roby and Scott, 2016a) non-elicited-response tasks: children who hear more cognitive terms more readily anticipate the behavior of a mistaken agent. Although additional research is needed to determine the causal mechanism behind this relationship, these findings are consistent with the notion that children who frequently engage in conversations about others’ thoughts and beliefs more readily attend to belief-relevant information within a scene and more quickly retrieve an agent’s false belief when necessary. These results suggest that parental use of mental-state language is related to false-belief performance prior to the preschool years, and that this relationship extends to non-elicited-response false-belief tasks (for related evidence see Johnson et al., 2007; Taumoepeau and Ruffman, 2008; Newton et al., in press).

Second, social factors should continue to facilitate false-belief understanding after the preschool years. Support for this prediction comes from several recent studies suggesting that elementary-school children’s personal use of mental-state language is correlated with their false-belief understanding, and that training accurate use of this language improves false-belief performance (e.g., Grazzani and Ornaghi, 2012; Lecce et al., 2014; Bianco et al., 2016). Recent work in our lab suggests that this association persists into adulthood: adults who use more mental-state language when describing social images are faster at accurately predicting the behavior of an agent in an avoidance false-belief task, suggesting that these individuals more readily attend to mental-state relevant information and are faster at retrieving mental states under time pressure (Roby and Scott, 2016b; see also Lecce et al., 2015).

## Conclusion

Here we have provided the first attempt at reconciling the well-established associations between social factors and preschoolers’ false-belief performance with the mentalistic claim that false-belief understanding emerges in infancy. We have outlined several ways that social experiences facilitate the use of mental- state concepts across the lifespan. Consistent with this account, a growing body of evidence suggests that social experiences contribute to false-belief performance both before and after the preschool years.

## Author Contributions

Both authors contributed directly and equally to the work and approved it for publication.

## Conflict of Interest Statement

The authors declare that the research was conducted in the absence of any commercial or financial relationships that could be construed as a potential conflict of interest. The reviewer EB and the handling Editor declared their shared affiliation, and the handling Editor states that the process nevertheless met the standards of a fair and objective review.
